# Collected data on displacement and vibration tests of layered floor assembly constructed by using glued timber trusses

**DOI:** 10.1016/j.dib.2026.113101

**Published:** 2026-07-23

**Authors:** Aarno Hatsala, Hüseyin Emre Ilgın, Niko Kinnunen, Juha-Pekka Luukkainen, Fanny Malmstedt, Sami Pajunen, Katja Rinkinen, Juho Sormunen

**Affiliations:** a Tampere University, Faculty of Built Environment, Tampere, Finland; b South-Eastern Finland University of Applied Sciences (XAMK), Savonlinna, Finland

**Keywords:** Glued timber truss, Floor vibration, Intermediate floors, Layered floor assembly

## Abstract

This dataset contains detailed measurements from a full-scale experimental investigation of layered intermediate floor systems constructed using glued timber trusses. The experimental programme followed a step-by-step construction sequence involving the incremental addition of typical building layers, including OSB sheathing, gypsum board ceilings, concrete topping, and lightweight partition walls. Static displacement tests were conducted under controlled loading using water tanks, with vertical displacements measured by digital dial gauges. Vibration response was assessed using heel-drop excitation applied at multiple locations and recorded with triaxial accelerometers. The dataset captures the progressive influence of individual construction layers on the global stiffness and vibration performance of the floor system under conditions representative of residential buildings. The data support reuse in serviceability assessments, comparative studies of floor assemblies, standardization and guideline development, and experimental research on lightweight timber structures. The accompanying documentation includes metadata, raw and processed measurement files, sensor and loading layouts, and visualization diagrams to enable independent analysis and interpretation. The dataset is associated with the related research article.

Specifications Table

**Table:** 

Subject	Engineering & Materials science
Specific subject area	Deflection and vibration behaviour of lightweight timber floors
Type of data	Table, Chart, Figure Raw, Processed
Data collection	The deflection and vibration data were measured in the laboratory using static loadings and heel-drop excitations. The measurements were carried out over numerous construction phases.
Data source location	Institution: South-Eastern Finland University of Applied Sciences (XAMK), Structural Engineering Laboratory, Finland.
Data accessibility	Repository name: Zenodo DOI: https://doi.org/10.5281/zenodo.20177655
Related research article	J. Sormunen, S. Pajunen, H.E. Ilgın, A. Hatsala, F. Malmstedt, J.-P. Luukkainen. Structural vibration analysis and design of glued timber truss intermediate floors. Engineering Structures, 357 (2026), 122502. https://doi.org/10.1016/j.engstruct.2026.122502

## 1 Value of the Data

• The dataset documents the static and dynamic response of a full-scale glued timber truss intermediate floor system tested under conditions representative of residential service loading. The staged experimental approach enables analysis of system behaviour as individual floor and ceiling layers are sequentially introduced.
• Researchers in structural engineering and timber construction can reuse the data to quantify the relative influence of OSB sheathing, gypsum board ceilings, concrete topping, and lightweight partition walls on floor stiffness and vibration response. The consistent instrumentation and testing procedures applied throughout all phases support direct comparison and reproducibility.
• The dataset includes static displacement measurements, raw time-domain acceleration signals, and processed frequency information from heel-drop testing.
• The data are relevant for structural engineers, researchers in timber construction, developers of numerical (e.g. FEM) models, standardization bodies, and practitioners involved in the design and assessment of lightweight floor systems. The dataset provides reference measurements for validation, benchmarking, and comparative analysis of floor vibration performance under serviceability conditions and phase-by-phase stiffness evolution.

## 2 Background

Lightweight timber and hybrid intermediate floor systems are increasingly used in residential and modular construction due to their low self-weight, material efficiency, and suitability for prefabrication. However, these systems are often governed by serviceability [1]. Because of the low mass, floor structures can be sensitive to vibration, deflection, and user comfort under normal use. Glued timber truss floors offer an efficient solution, combining open-web geometry with adhesive connections. This allows material-efficient load transfer and integration of building services. Compared to more established systems such as CLT and timber–concrete composite floors, their full-scale behavior is still less documented. In particular, vibration and deflection response depend on the interaction between trusses, sheathing, ceilings, added mass, and non-structural elements. Systematic full-scale datasets covering different construction stages are limited. The dataset [2] was generated within the PuuHyVä project and is linked to [3]. It includes static displacement measurements, raw acceleration time histories, processed vibration results, and documentation for different construction phases. The data can be used for validation of numerical models, comparison of floor configurations, and serviceability assessment of lightweight timber floors.

## 3 Data Description

### 3.1 Drawings and deflection measurements

The dataset contains geometric documentation of the test structure together with static deflection measurements collected during the staged construction and loading of the glued timber truss floor system. The dataset includes detailed drawings of the glued timber trusses, measurement point layouts, and other relevant structural details required to interpret the displacement measurements.

**Fig. 1:**
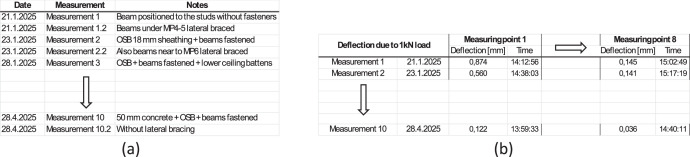
(a) Schematic overview of the phased construction and measurement sequence used for static deflection testing. (b) Example structure of a deflection measurement dataset, illustrating the organization of numerical values in the accompanying Excel sheets.

Static deflection data, measured using digital dial gauges, are provided as numerical values recorded at predefined measurement points (MPs) and compiled in Excel files. The measurement configuration and loading sequence are illustrated in Fig. 1, which presents also an example of the deflection measurement documentation.

One measurement included a controlled variable loading test using a water-filled IBC tank. During this phase, the tank was placed first at measurement point MP2 and subsequently at a location between MP4 and MP5 at mid-span, with the tank weight monitored using a scale positioned beneath it. The tank was filled and emptied continuously through a hose, and the applied load and corresponding vertical deflections were recorded at 30 s intervals. Residual deflections following unloading were also documented. The deflection data associated with this variable loading phase are provided in a separate Excel worksheet.

In addition, vertical displacements at two measurement points were monitored during the concrete casting phase and for a subsequent observation period of six weeks. These time-dependent displacement measurements are included in a dedicated worksheet within the dataset.

### 3.2 Vibration measurements

**Fig. 2:**
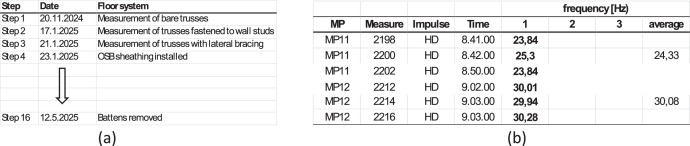
(a) Overview of vibration measurement stages linked to the phased construction sequence. The dynamic step numbers represent chronological measurement stages and do not correspond directly to construction phases. (b) Example of processed vibration data obtained from a single heel-drop test, illustrating the type of frequency-domain information provided in the dataset.

Additionally, the dataset contains vibration measurement data obtained from heel-drop tests performed during multiple construction phases of the glued timber truss floor system. The dataset comprises both processed vibration characteristics and raw acceleration time histories, enabling detailed analysis of transient and frequency-domain floor response. An example of the vibration measurement configuration and typical processed results is shown in Fig. 2.

Processed data are provided in Excel file containing the identified fundamental frequencies for each measurement step. The Excel sheets are named according to the test date, allowing the vibration characteristics to be linked directly to the corresponding construction stage.

In addition to the processed results, vibration measurement data includes raw acceleration data recorded during the heel-drop tests. These raw data are provided as ASCII files with the file extension *.dat* and may be used to reproduce the transient vibration response during and after impact. Each measurement consists of one or more data files, depending on the duration of the recording. Longer recordings are divided into sequential packets, each containing a fixed number of samples.

The naming convention of the raw data files follows the format *devXXX_YYY_Z.dat*, where *XXX* denotes the measuring device number, *YYY* the measurement identifier, and *Z* the packet number within the corresponding measurement. For example, the file *dev004_839_3.dat* represents the third data packet recorded by device 4 during measurement 839.

Each raw data file contains time-series acceleration data sampled at a constant frequency of 1000 Hz. Each data line includes the running sample number, the elapsed time from the start of the measurement expressed in microseconds, and the measured accelerations in the x-, y-, and z-directions. Acceleration values are reported in milligravity units (mg). In the adopted coordinate system, the z-direction corresponds to the vertical axis; consequently, a value of approximately 1000 mg represents gravitational acceleration. *For example, a data line of the form* 5016t4937471×20.096y3.266z1001.596 *indicates sample number 5016, an elapsed time of 4.94 s from the start of the measurement, and acceleration components in the x-, y-, and z-directions expressed in milligravity units (mg).*

The overall organization of the dataset and the relationship between phases, measurements, and files are described below.

### 3.3 Dataset structure and organization

To support reuse, the dataset is organized into folders separating static deflection data, vibration data, and supporting documentation as summarized in Table 1. The data are linked to the phased construction sequence of the floor system so that after each construction phase certain static and vibration measurements are carried out as reported in Table 2. The dynamic vibration numbering represents chronological measurement steps and does not directly correspond to construction phases; multiple measurements may be performed within a single construction phase, and the total of sixteen steps therefore exceeds the number of construction stages. Steps 13–16 correspond to dismantling stages. A README file is included in the repository to improve the usability of the dataset.

**Table 1:** Overview of dataset contents.

File	File type	Description	Units	No. of files
Deflection data	.xlsx	Static displacement measurements	mm	1 file
Vibration data (processed)	.xlsx	Identified fundamental frequencies	Hz	1 file
Vibration data (raw)	.dat	Acceleration time series	mg, µs	1 zip-file, 1430 files
Metadata	.pdf	Drawings, measurement points	–	7 files
Documentation	.pdf	README-file		1 file

**Table 2:** Relationship between construction phases, static measurement IDs, and dynamic vibration measurement steps.

	Construction phases	Static measurements	Dynamic measurement steps
1	Placement of truss beams on supporting walls	1, 1.2	1, 2,3
2	Installation of OSB sheathing	2, 2.2	4
3	Installation of lower ceiling battens	3	5
4	Assembly of the bottom OSB sheathing	4	6, 7, 8
5	Casting the concrete topping layer	5, 6, 7, 7.2	9,10,11
6	Installation of internal partition walls	8	12
7	Dismantling in reverse order	9, 10, 10.2	13, 14, 15, 16

Note: Dynamic measurement steps are chronological identifiers and do not correspond one-to-one to construction phases. Several measurements may be conducted within a single phase, and Steps 13–16 represent dismantling stages of the floor assembly.

## 4 Experimental Design, Materials and Methods

The dataset was obtained from full-scale experimental testing of a timber truss intermediate floor assembly with approximate plan dimensions of 10.5 m × 5.5 m. The experimental programme followed a phased construction protocol designed to systematically vary the mass and stiffness of the floor system while maintaining consistent measurement conditions. The programme included multiple construction, loading, and dismantling stages, during which static and dynamic measurements were carried out. The structural configurations, including support conditions of the floor system, are documented in the drawings provided in the dataset repository. The experiments were conducted under controlled laboratory conditions with temperature maintained at 20 ± 2 °C and relative humidity at 65 ± 5 %. All materials were stored under these conditions for at least two months prior to testing.

**Fig. 3:**
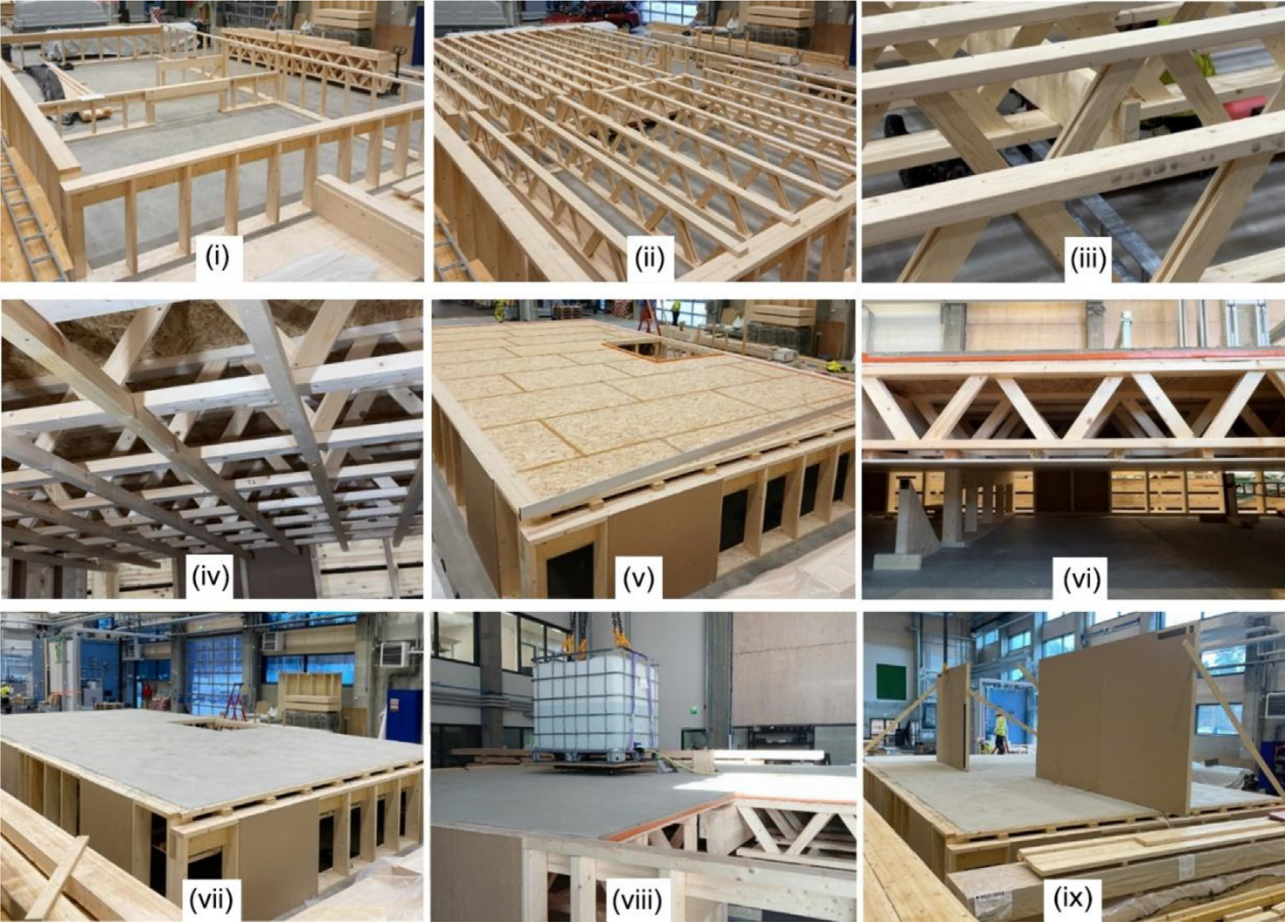
Schematic phased construction sequence of the full-scale timber truss floor mock-up corresponding to the data acquisition stages: (i) supporting structures; (ii–iii) TK-beams; (iv) ceiling battens; (v) OSB sheathing; (vi) bottom sheathing; (vii) concrete topping; (viii) IBC load tanks; (ix) partition walls.

The construction phases included the sequential installation of 18 mm OSB/3 sheathing, ceiling battens and gypsum boards, added permanent masses, and a 50 mm concrete topping with strength class C30/37, for which vibration measurements were taken a few days after casting and deflection was monitored from the moment of casting over a six-week period. The main structural components consisted of glued timber trusses with C24 timber in the top and bottom chords, while diagonals and secondary members were made of non-graded sawn timber. Wall frames and bracing elements were also made of C24 timber. Additional phases involved serviceability loading using water-filled IBC containers, installation of lightweight partition walls, application of sand layers, a reference configuration, and selective removal of layers. The phased construction sequence corresponding to the data acquisition stages is illustrated schematically in Fig. 3.

**Fig. 4:**
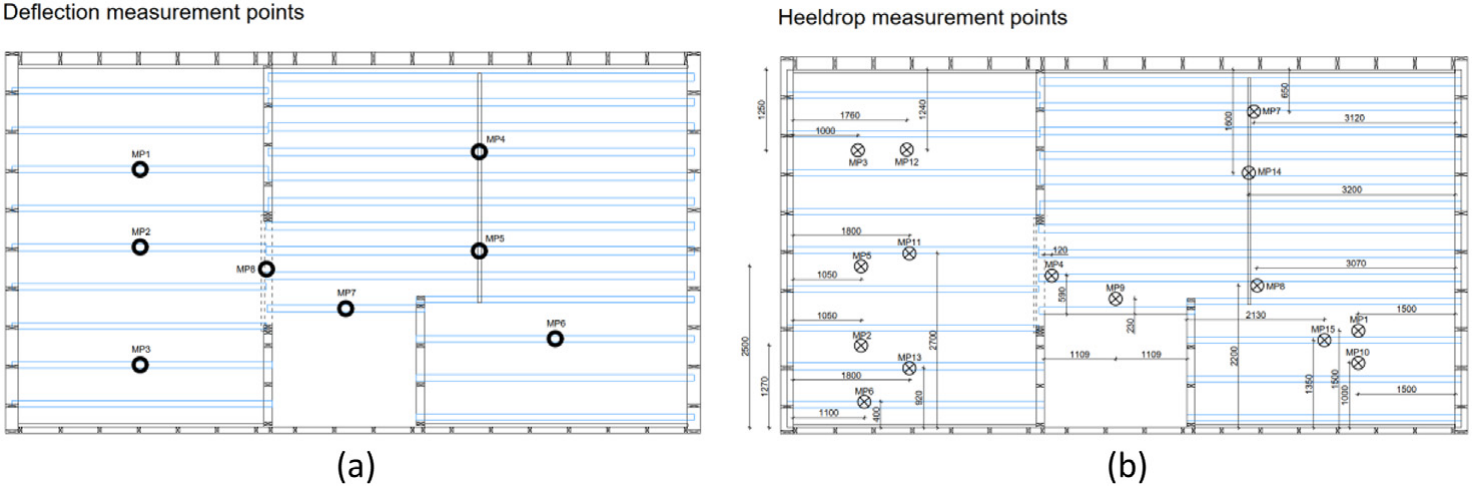
(a) Measurement points used in static tests. (b) Accelerometer measurement points used in the vibration tests.

Static deflection measurements were conducted using three Mahr MarCator 1086 R digital dial gauges installed beneath the floor structure. The gauges have a measuring range of 12.5 mm, an error limit of 0.004 mm, and a repeatability of 0.001 mm, and were mounted using magnetic stands (Fisso Strato Line XS-13). Deflections were recorded at predefined measurement points located at mid-span of the beams, as shown in Fig. 4a. The measurement accuracy is therefore considered to be on the order of 0.001 mm, based on the specified repeatability and confirmed by an internal repeatability check. No additional calibration was performed during the study. Applied loads were positioned at each measurement point in turn, and the corresponding vertical displacements were recorded digitally. Measurements were taken once per phase and measurement point using a test person with approximately constant mass of 94 kg, and the results were scaled to a reference load of 1 kN. The measurement procedure was repeated after each construction or loading modification, and all deflection data were documented in Excel format. In addition to standard loading using a test person, a variable loading test was performed on the completed floor assembly using a water-filled IBC container (static measurement 7.2 in Table 2). The load was controlled by adjusting the water volume in the tank. Deflections were recorded continuously during filling and emptying of the tank, as well as at constant load levels after the structure had settled.

Dynamic response data were obtained using heel-drop excitation in combination with triaxial accelerometer measurements. The measurements were conducted using a measurement system developed by Doftech Oy. The sensor orientation was corrected after installation using a rotation matrix to ensure that the vertical (z-direction) response was accurately captured, and the measurement device was rigidly attached to the structure to follow the structural motion during testing. Acceleration time histories were recorded at predefined measurement points (MP1–MP15) and excitation locations illustrated in Fig. 4b, using a sampling frequency of 1000 Hz. This value was selected based on EN 16,929:2018, which recommends that the sampling frequency is at least ten times the highest evaluated natural frequency of the structure. The selected frequency ensures sufficient resolution for identifying the dominant vibration response and enables evaluation of derived quantities such as velocity and displacement. For each measurement, the accelerometer was positioned at the selected measurement point, and the heel-drop excitation was applied at a distance of approximately 500 mm from the sensor. At each measurement point, at least three consecutive measurements were performed, and the excitation amplitude was controlled to remain within approximately ±1 g. Raw acceleration signals were retained for all tests, and processed datasets were subsequently used to identify fundamental vibration frequencies for each construction phase and measurement location. Frequency identification was performed using frequency-domain analysis based on the Fast Fourier Transform (FFT). The vertical acceleration time histories were recorded at a sampling frequency of approximately 1 kHz, and each complete measurement record was treated as an individual analysis window. Before transformation, the mean acceleration was removed. The FFT was applied without additional window weighting (rectangular window), and no filtering, overlapping windows, or FFT averaging were used. Because the record lengths varied, the frequency-bin spacing was determined separately for each measurement as Δ*f* = *f*_s_/*N* = 1/*T*, where *f*_s_ is the sampling frequency, *N* is the number of samples, and *T* is the record duration. The fundamental frequency was identified from the dominant peak in the low-frequency spectrum. When multiple peaks were present, the selected frequency was required to occur consistently across repeated measurements and to be compatible with the expected global floor response. Isolated peaks that were not repeatable between consecutive measurements were excluded.

To assess the reliability of the measurements, comparative tests were conducted using two accelerometers attached to a steel plate (mass 3.5 kg) located at the distance of 500 mm from the heel-drop excitation point on the investigated structure. Measurements were repeated throughout the campaign. Based on 56 paired measurements, the correlation coefficient was 0.999 and the median difference in identified frequencies was 0.095 Hz. No systematic drift between the sensors was observed. Repeatability was assessed based on repeated measurements collected over multiple test days. At each measurement point, 3–5 consecutive heel-drop tests were performed, and representative values were determined after excluding clear outliers. The standard deviation of repeated measurements was evaluated for each test series and subsequently averaged for each measurement day. The resulting average standard deviations ranged from 0.089 Hz to 0.941 Hz, with a mean value of 0.283 Hz. For measurements conducted after concrete casting, the corresponding mean standard deviation was 0.143 Hz. Based on these results, the uncertainty of the identified fundamental frequency can be considered to be within approximately ±0.73 Hz for the full dataset and ±0.37 Hz for measurements after concrete casting. These values reflect the combined influence of excitation variability and measurement noise.

## Limitations

This dataset is based on a single full-scale glued timber truss floor specimen and therefore reflects the behaviour of one specific structural configuration rather than the variability of nominally similar systems. All measurements were conducted under controlled laboratory conditions, which enabled consistent instrumentation and staged construction but do not fully represent in-situ conditions, including structural continuity, workmanship variability, service penetrations, occupant use, or environmental effects. Dynamic measurements were based on heel-drop excitation, which introduces inherent variability due to differences in excitation conditions. In addition, frequency identification may be influenced by closely spaced modes and broad spectral peaks typical for layered timber floor structures. The adopted measurement methodology also imposes limitations, as static deflections were measured only at discrete points and vibration measurements were limited to selected sensor locations, which may not capture all local or torsional responses. These factors define the boundary conditions for using the dataset in benchmarking and model validation. The dataset is most directly applicable to floor systems with similar configurations, while its application to other timber floor typologies requires case-specific validation. The dataset does not capture long-term behaviour, including aging, creep, repeated loading, or environmental variations. Additional notes on individual measurements, including cases affected by increased noise or unsuccessful test execution, are documented within the dataset and should be considered when interpreting specific results.

## Ethics Statement

The authors have read and followed the ethical requirements for publication in Data in Brief and confirm that the present work does not involve human subjects, animal experiments, or any data collection from social media platforms.

## CRediT Author Statement

**Aarno Hatsala**: Investigation; **Hüseyin Emre Ilgın**: Writing – original draft, Writing - Review & Editing; **Niko Kinnunen**: Investigation, Conceptualization; **Juha-Pekka Luukkainen**: Resources, Project administration; **Fanny Malmstedt**: Investigation, Conceptualization; **Sami Pajunen**: Validation, Supervision, Writing – original draft, Writing - Review & Editing; **Katja Rinkinen**: Investigation, Conceptualization; **Juho Sormunen**: Methodology, Validation.

## Data Availability

ZenodoCollected data on displacement and vibration tests of layered floor assembly constructed by using glued timber trusses (Original data).
